# Examining the Moderating Role of Reasons in Masstige Luxury Buying Behavior

**DOI:** 10.3390/bs14010067

**Published:** 2024-01-19

**Authors:** Ayse Sedef Uluturk, Umut Asan

**Affiliations:** Department of Industrial Engineering, Istanbul Technical University, Macka, 34367 Istanbul, Türkiye; asanu@itu.edu.tr

**Keywords:** behavioral reasoning theory, reasons, masstige jewelry consumption, moderator effect, partial least squares-structural equation modeling

## Abstract

This study proposes a new model derived from Behavioral Reasoning Theory (BRT) to examine the purchasing behavior of masstige jewelry consumers. The suggested model provides a more comprehensive understanding of the determinants of purchasing masstige products by considering values and reasons in addition to the global motives and intention. The study also examines, for the first time, the moderating role of reasons. It explores how reasons may strengthen or weaken the impact of perceived values on global motives. The proposed model was empirically tested using partial least squares-structural equation modeling (PLS-SEM) with survey data on the consumption of masstige jewelry brands in Türkiye. To provide a more complete understanding of the moderating role of reasons, submodels were constructed for different value–reason combinations. The results demonstrate notable differences in the goodness-of-fit measures between the moderated and non-moderated models. Specifically, reasons contributed to enhanced explanations of global motives and intention, both directly and indirectly. However, not all submodels yielded significant results in terms of the moderator effect. Thus, the empirical tests supported the hypotheses regarding the moderating effect only partially. Overall, the current findings further extend the existing frameworks and provide valuable insights into masstige jewelry purchasing behavior, which can be used by marketers to develop more effective strategies.

## 1. Introduction

The luxury product market has experienced a rapid growth in the last twenty years in the world. According to the research of Bain and Company (2023), the total personal luxury product market reached EUR 353 billion in 2022 and is estimated to reach EUR 540–580 billion by 2030 [1]. On the other hand, reasons such as the economic stagnation experienced in America and Europe in the 1990s, the cessation of population growth in Europe, and the growth of a middle class who wanted to reach luxury caused the need for change and expansion in luxury brands [2]. With these recent structural and cultural changes in the capitalist markets, luxury brands that have undergone change have become accessible to the masses [3,4]. Many studies have defined this change using different terms, like the ‘democratization of luxury’ or ‘luxury for the masses’ or ‘masstige (mass-prestige)’, which is the focal point of this study. While the democratization of luxury is a frequent topic of discussion in industry reports, the academic literature remains notably unexplored in this realm [5].

With the growth and expansion of the masstige luxury market, the analysis of luxury consumer buying behavior has become even more important. The jewelry market especially, which is addressed in this study, is growing and spreading day by day with the emergence of masstige jewelry brands. However, research examining the purchasing behavior of jewelry consumers, especially for masstige products, is limited, which underscores the importance of this study. 

Luxury goods, which are accepted as high-involvement products due to their high price, rarity, and complicated nature, require detailed research and knowledge. The consumer is more likely to choose the decision alternative that is supported by strong reasons and aligns with his/her values, as they guide the reasoning and selection of decision alternatives. Reasons serve as context-specific factors that consumers use to justify and defend their judgements or intentions that affect their behavior [6]. Values serve as a framework for what is important and meaningful to consumers. Therefore, incorporating the concepts of ‘reason’ and ‘value’ into the modeling of (masstige) jewelry purchasing behavior will provide a more comprehensive explanation of this behavior. Only a limited number of studies rooted in Behavioral Reasoning Theory (BRT) have investigated the influence of values and context-specific reasons on consumer behavior. Specifically, reasons are modeled as mediators between beliefs/values and global motives, where it is assumed that the way individuals process their belief and value information directly influences the reasons they provide for explaining their behavior [6]. Despite its advantages, BRT remains significantly underexplored [7]. Only a few recent studies, including those by Sivathanu [8,9], Pillai and Sivathanu [10], Gupta and Arora [11,12], Claudy et al. [13,14], and Ryan and Casidy [15], have tested different hypotheses stemming from both the original and modified versions of the BRT theory. However, none of these models have been examined within the realm of luxury consumption (see [7] also).

In luxury consumption, the reasons are generally based on traditions, social culture, and self; therefore, they are considered as psychological structures believed to be relatively more stable and have contextual characteristics (see [16]). In addition, reasons may not necessarily come after values temporally (see [17]). Reasons may strengthen or weaken the influence of perceived values on global motives. For example, consumers who share identical values may develop different attitudes due to their varying reasons for purchasing jewelry. Therefore, it is essential to establish a model that examines the moderating role of reasons. Sahu et al. [7] point out in their review of BRT that only a few studies have examined the impact of moderating variables (especially between values and attitudes), indicating a significant research gap in the existing literature on this topic (see also [6,14]).

Based on the above discussions, three research gaps in the current literature will be addressed in this study. First, further research is required to model consumer behavior in the growing masstige luxury market, particularly in the jewelry segment. Second, previous research has shown that values and reasons are important in explaining consumer behavior, but there is limited research on the specific values [18] and none on reasons that underlie masstige jewelry consumption. Third, a deeper exploration of the roles of reason in luxury consumption is needed. Specifically, the interaction of values and reasons and their potential impact on global motives have not been examined yet. Addressing these research gaps will not only uncover the key drivers and motivations behind consumer choices in the masstige jewelry sector but will also extend the theory of behavioral reasoning. This will lead to more informed decision-making, targeted marketing efforts, and a better understanding of consumer motivations in the context of luxury consumption.

To narrow these gaps, this study proposes a modified model derived from BRT for the examination of purchasing behavior of luxury consumers. The proposed model is designed specifically for the consumption of masstige jewelry, for which the concepts of BRT and reasons have not been considered before. The proposed model examines the impact of values and reasons on global motives and intentions, aiming to provide a more comprehensive understanding of purchase behavior. Given consistent context-specific reasons, they are assumed to act as potential moderators between perceived values and global motives. In other words, reasons may strengthen/weaken or affect the direction of the causal relationship between the perceived values and global motives. In order to evaluate the proposed model and test the hypotheses, partial least squares-structural equation modeling (PLS-SEM) was employed using survey data on the consumption of brands in the masstige jewelry market in Türkiye.

The key contributions of this empirical study can be outlined as follows:

- By analyzing consumer behavior in the growing masstige luxury market in Türkiye, particularly in the jewelry segment, the proposed model enriches the literature, where research is very limited.
- A new model derived from BRT is introduced to examine the determinants of purchasing behavior of masstige jewelry. The proposed model offers a more comprehensive understanding of consumers’ intention to purchase masstige products by considering values and reasons in addition to the global motives and intention.
- This study, for the first time, explores how reasons may strengthen or weaken the impact of perceived values on global motives. It provides a deeper understanding of the role of reasons in luxury consumption.

The rest of this paper is organized as follows. First, a review of the literature on luxury and masstige luxury purchasing is provided. The subsequent section introduces the proposed research model and outlines the research hypotheses. Section 4 presents the methodology used in the empirical study, while the subsequent section provides the results of the PLS-SEM analysis and the corresponding findings. In the final section, contributions, limitations, and further research opportunities are presented. 

## 2. Luxury and Masstige Consumption

The term ‘masstige’ has its roots in the luxury concept, which originates from the Latin word ‘luxus’ and means ‘extras of life’ or ‘extravagant life’ [19]. Until the fourteenth century, the common people associated negative meanings with the concept of luxury. In the following centuries, luxury was seen as an indicator of the noble class or of social class distinction with the emergence of the bourgeoisie in Europe [20]. Today, luxury brands and products are often associated with desire, pleasure, comfort, exclusivity, status, and identity. 

In the academic literature, the concepts of luxury and luxury brands lack a universally accepted definition [21]. Researchers employ the term ‘luxury’ to characterize the highest tier of prestigious and high-status brands, as noted by Vigneron and Johnson [22]. According to Vickers and Renand’s [23] definition, luxury products serve as symbols of both personal and social identity. Consumers believe that luxury brands bring dignity to their owners and meet functional and psychological needs; therefore, the concept of luxury consumption is defined as ‘conspicuous consumption’ [24]. According to another definition, ‘luxury brands’ are expensive, high quality, non-essential offers that have symbolic or emotional value and are perceived as rare, unique, prestigious, and authentic by the consumer [25]. 

The following subsections provide an overview of the masstige luxury concept and related works.

### 2.1. Democratization of the Luxury

When the luxury product market is examined, it is evident that the market has experienced a rapid growth worldwide in the last thirty years. Despite declining sales due to the COVID-19 pandemic, in 2021, the luxury goods industry underwent a rapid recovery characterized by a V-shaped pattern [1]. Despite the worsening global macroeconomic indicators, the personal luxury market achieved a retail sales volume of EUR 353 billion in 2022. [1]. Bain and Company predict that the personal luxury goods market has the potential to achieve a volume of EUR 540–560 billion by 2030, assuming a consistent annual growth rate of 6-8% [1].

Parallel to these developments, economic growth has created a middle class (especially in emerging markets, such as China, India, and Türkiye) with great market potential [26,27,28]. Companies are now venturing into non-traditional approaches to market luxury products and services, offering consumers the allure of status, indulgence, and an exceptional experience. In recent years, the definition of ‘luxury’ has evolved, giving rise to the concept of ‘new luxury’ [29], which encompasses notions of affordability, wider market reach, and accessibility within the mass market [4].

With these recent changes, luxury brands have become accessible to the masses [3]. Vigneron and Johnson [22] highlight a significant shift in the last two decades, where brands expanded their marketing efforts from solely targeting the wealthiest consumers to also encompassing middle-class consumers. This was achieved through new product lines, new brand launches, and brand extensions. This development is commonly termed as the ‘democratization of luxury’ [30]. With the democratization of luxury, many companies have expanded their product range, and they have become accessible both in terms of price and geography. Firms such as Toyota, Apple, and Honda appear to be adopting a new marketing approach aimed at enticing aspiring middle-class consumers seeking prestige, but at a more accessible price point [31]. Luxury brands like Versace, Alexander Wang, Balmain, and Marni, among others, have engaged in licensing or collaborative partnerships with high street retailers such as H&M. This collaboration aims to offer a more affordable rendition of their luxury items, catering to a broader middle-class consumer base through mass production [32]. Capsule collections from haute couture designers elevate the status of ready-to-wear clothing or masstige brands, enabling the middle class to access signature products. The collaborations between Arzu Kaprol and Network, Ozlem Suer and Atasay, and Dilek Hanif and Koton serve as illustrative instances within Türkiye.

Three different paths have been followed in the democratization of luxury [33]. ‘Accessible Superpremium’ products have higher quality or taste than other products in their category and are priced above other products in the category, but these products are still accessible to mid-range consumers [34]. For example, Starbucks and Belvedere Vodka are priced 40% higher than similar products [35]. ‘Old-luxury brand extensions’ are well-established luxury brand extensions, which are affordable products of traditional luxury brands. This new generation of luxury goods refers to products that are not unique but are produced in limited quantities. These products achieve the luxury label because of the design, the aura, or the additional services the brand creates. In this new definition of luxury, consumers are more concerned with the image of the brand than the product itself. In the list of traditional luxury brands that have expanded their brands, there are Mercedes-Benz, Ermenegildo Zegna, Tiffany, and Burberry, which offer economical products along with traditional products [34]. ‘Masstige’, as the third path, creates a niche market between mass products and traditional luxury brands [36]. As a fusion of ‘mass’ and ‘prestige’, ‘masstige’ brands offer goods that exhibit superior quality, elegance, and desirability within their category, yet remain within an affordable price range [34]. In other words, the ‘masstige’ concept refers to a phenomenon wherein premium or high-value products are marketed to a broad customer base by cultivating a sense of widespread prestige, all while maintaining consistent pricing [37]. They provide the consumer with emotional benefits such as prestige, self-actualization, or group membership. While traditional luxury items retain their prestige by emphasizing premium pricing and exclusivity [38,39], masstige products adopt a mass-targeting approach with relatively lower pricing and limited accessibility, all while upholding brand prestige. Couch, Miu Miu, and Armani Exchange are examples of mass-prestige brands [35]. Louis Vuitton, on the other hand, has developed masstige products in the jewelry industry [36]. In addition to prestige and affordability, masstige goods must exhibit superior quality, multifunctionality, and prolonged utility. Particularly in developing countries characterized by extensive mass markets, the significance of the masstige marketing strategy is widely acknowledged. The masstige strategy achieves success by striking a harmonious equilibrium between differentiation through prestige and a justifiable premium. Masstige marketing represents a phenomenon where pricing is regarded as a composite outcome of product, promotion, and placement strategies [31].

As the masstige luxury market expands, understanding the buying habits of masstige consumers has become even more important. The jewelry sector, which is a main branch of luxury consumption, is also growing and becoming widespread day by day, especially with the emergence of masstige jewelry brands, which is the main focus of this study. Jewelry is highly valued worldwide due to the intrinsic and symbolic value it offers to consumers [40]. Consumers purchase jewelry not primarily for financial gains, but rather as an emotional investment [41]. In the past, buying jewelry was limited to specific occasions and weddings, but nowadays, people are buying jewelry as a means of self-expression and to enhance their personal style (see also [41]). Academic studies on the buying behavior of jewelry consumers and masstige luxury are limited, which makes this study more valuable.

### 2.2. Purchasing Behavior of Luxury and Masstige Consumers

Previous studies in the luxury product market have predominantly examined the meaning and measurement of luxury and masstige (e.g., [3,42]), values and motives in purchasing luxury and masstige goods (e.g., [21,43,44]), determinants of luxury buying behavior [45] (for a comprehensive review refer to [46]), and the management of masstige and luxury brands (e.g., [30,37]). Only the studies related to the current work will be mentioned here in more detail. 

Motivations for luxury consumption have to be thoroughly examined in order to understand the consumers’ perceptions of luxury brands, which is a prerequisite for successful brand positioning and market segmentation. Previous studies have focused on defining the dimensions of the luxury value concept. Wiedmann et al. [21] explored luxury values from the standpoint of consumers and identified four key dimensions: functional value, individual value, social value, and financial value. Likewise, Berthon et al. [47] presented a three-dimensional model, including experiential, symbolic, and functional values. 

On the other hand, Paul [42] introduced a theoretical model and scale to operationalize mass prestige. He conceptualized ‘masstige value’ as a substitute for brand equity and developed a scale and index, serving as a benchmark measure for evaluating and comparing the ‘Masstige value’. Ghimire et al. [48] studied the value dimension of masstige brands based on the Woodruff’s [49] customer value determination framework. They determined that Indian customers believed that masstige brands have the highest functionality and that they provide self-directed image value. They also proposed that the masstige brands possess qualities of superiority, fashion-forwardness, trendsetting, self-customization, and innovation. Kim et al. [44] investigated the values conveyed by advertising messages from luxury and masstige fashion brands. Shared implicit values in these ads encompass exclusivity, sophistication, authenticity, achievement, and pride. Symbolic interpretations and motifs commonly associated with masstige brand advertisements include seasonality, allure, and rejuvenation. In another recent study, Kumar et al. [4] introduced the mass–luxury continuum to categorize product or service brands in relation to mass prestige. They also outline the steps for creating a masstige brand, focusing on the marketing mix.

Various studies have focused on different determinants for luxury consumption. In an empirical study, Jamal and Goode [50] examined the criteria used when purchasing precious jewelry. Their study emphasized the importance of product category expertise, brand familiarity, and brand awareness in product assessment. Zhan and He [51] suggested that culture has an impact on the attitudes and buying intentions of luxury consumers. Their study focused on Chinese consumers and found that collectivist cultures are predominantly motivated by social needs. Additionally, they identified value consciousness and the desire for uniqueness as further key psychological factors impacting consumer buying behavior in China. The authors also analyzed the moderating role of consumer knowledge. They claim that consumers are less likely to use the best-known, popular luxury brands to express their uniqueness when consumer knowledge increases. Granot et al. (2013) conducted exploratory research focusing on consumer perceptions of masstige brands, considering factors like conspicuousness, style, signaling, self-indulgence, exploration, and quality, as well as their impact on purchase decisions. Loureiro and Araújo [18] examined the influence of individual and social luxury values on customer attitudes, perceived behavioral control, and subjective norms, as well as how these factors, along with past experiences, affect intentions to recommend and pay a premium for luxury clothing in the Brazilian market. The results indicate that individual values positively affect intentions, while social values positively influence subjective norms but negatively affect perceived behavioral control. Past experience does not notably impact intentions. Hennigs et al. [52] demonstrated the significant connection between perceptions of luxury brand value and important outcomes like purchase intention, recommendations, and willingness to pay. Riley et al. [53] investigated the influence of perceived value in the connection between perceived fit, brand attitude, extension attitude, and consumers’ purchase intent for downscale vertical extensions of premium and luxury brands in the automotive and footwear markets. Their findings demonstrate that perceived value acts with a partial mediating role in the relationships between brand attitude and extension attitude with purchase intention. In another study, Mason et al. [54] examined the influence of the Lipstick effect, employing income as a moderating variable on the relationship between the primary aspects of service quality and behavioral intentions in a new luxury context. Loureiro et al. [55] analyzed how involvement, perceived self, social values, and desire are related to consumer engagement in the fashion luxury context (masstige brands). They also explored the role of past experience as a moderator in the relationship between consumer engagement and subjective well-being. In a more recent study, Zhang et al. [45] explored the factors influencing Chinese consumers’ purchase intentions for luxury goods by extending the TPB. The findings indicate that both perceived behavioral control and subjective norms had notable and direct effects on consumers’ purchase intentions. Furthermore, prior experience with luxury goods purchases also directly influenced consumers’ intentions to buy such products.

Review of the literature reveals that empirical studies examining the determinants of luxury/masstige purchasing behavior and their influence are rather limited, especially in the context of jewelry brands and products. The suggested models have neglected the role of ‘reasons’ in the motivation mechanism. This study aims to address this gap by examining the role of reasons and their interaction with values to explain the buying behavior of masstige luxury products.

## 3. Conceptual Background and Hypotheses

In this section, the research model derived from BRT will be presented to examine luxury product purchasing behavior. The proposed research model includes perceived values, global motives, and intentions that predict the behavior, as well as the reasons that lead consumers (not) to purchase. While perceived values, global motives, and intentions are interconnected by mediating effect, reasons have a moderating effect between perceived values and global motives. The proposed model is specifically designed for luxury consumption, where the BRT and reasons have not been used before. The concepts and relationships in the proposed model are explained in the context of masstige luxury consumption.

### 3.1. Concepts

Before presenting the proposed research model, the concepts involved in this model and their relationships will be explained.

#### 3.1.1. Perceived Value

‘Value’, as one of the most common concepts in the social sciences, may take on different meanings. In the field of marketing, the concept of value is regarded as one of the key concepts in understanding and predicting consumer behavior [56]. Luxury value perception and consumer motivation, which explain why consumers prefer certain products and avoid some products in the constantly and dynamically growing luxury consumption market, serve as guidelines for marketing decisions. 

The perceived value of a luxury product is associated with the broad concept of luxury, such as pleasure, rarity, high price, excellent quality, and aesthetic beauty. The luxury product consumer aims to meet these needs by purchasing luxury products. Researchers working in the field of luxury consumption have suggested different definitions of value. According to Wiedmann et al. [57], luxury value lies in individual (such as self-identity, hedonism, materialism) and social (such as prestige, conspicuousness) as well as in financial (such as investment, price) and functional (such as usability, quality, uniqueness) aspects. Vigneron and Johnson [58] identified five different perceived values that affect the purchasing process of consumers seeking prestige: convenience value, uniqueness value, social value, emotional value, and quality value. According to Shukla [59], the definition of luxury value includes conspicuousness, prestige, hedonism, materialism, uniqueness, and price–quality perception. 

In this research study, perceived values were primarily adapted from Wiedmann et al.’s [57] conceptual framework explaining consumers’ perception of luxury value. This framework provides a basis for effectively establishing, promoting, and assessing luxury brands or products across different cultures and is appropriate to reflect the cognitive and emotional value aspects of the jewelry consumers based on industrial experience. The perceived values used in this study include self-identity, hedonism, materialism, conspicuous consumption, prestige, accessible price, and quality. Among these constructs, accessible price was originally suggested in this study because of its importance in masstige consumption, and the description of each value construct has been adapted to align with the concept of masstige luxury. The descriptions of these perceived values are provided in Appendix A. 

#### 3.1.2. Reason

Reasons are context-specific factors that individuals use to explain their predicted behavior. People use reasons to make sense of the world as well as justify their behavioral choices, which helps them to avoid feeling uncomfortable or inconsistent [11,14]. According to BRT, when the individual has strong reasons for or against a behavior, he/she will form positive or negative evaluations of this behavioral alternative. The theory also indicates that the individual often favors the behavior with the largest set of verifiable and defensible reasons [6]. 

Reasons are important factors that affect jewelry buying behavior. Positive reasons can support, accelerate, and strengthen individuals’ decision to purchase jewelry, while negative reasons can negatively affect and weaken their purchasing behavior. In this study, only the factors that are assumed to positively affect jewelry purchasing behavior are discussed to not complicate the structure of the research further and to focus on the moderator effect of reasons. 

#### 3.1.3. Global Motives

Global motives basically include attitude, subjective norms, and perceived behavioral control factors. The first global motive attitude represents an individual’s favorable or unfavorable assessment of the relevant behavior. It is based on the consequences that are expected to be associated with the behavior [60]. 

Subjective norm indicates the perceived societal influences encouraging or discouraging a specific behavior. It is rooted in the perceived normative expectations of important referent individuals or social groups, like family, friends, relatives, co-workers, or experts in the relevant field [60,61].

The third global motive is the perceived behavioral control, the level of control (such as knowledge, ability) that individuals feel while performing a behavior [60,62]. It signifies an individual’s confidence in one’s capability to perform a specific behavior, influenced by one’s beliefs about the presence or absence of factors that aid or impede performing the behavior. Individuals perceive that they have behavioral control if the resources and opportunities they think they have are high and the difficulties they foresee are few [61].

It is worth noting that attitude, subjective norm, and behavioral control are assumed to be conceptually independent of each other. However, the relationship between these determinants is often not orthogonal [62]. All these antecedent factors play a mediating role between values and intentions [62], which will be explained later in detail.

#### 3.1.4. Intention

Intention, which is the main factor of behavioral intention models, is the level of the individual’s desire and effort to exhibit a certain behavior. Strong intentions will increase the likelihood of the behavior occurring [62]. While intention is the primary determinant of behavior, it is influenced by global motives. In other words, intention acts as a mediator for the impact of subjective norm, attitude, and perceived behavioral control on behavior [63]. 

### 3.2. Proposed Research Model and Hypotheses

The hypotheses presented in the research model have their foundations in behavioral intention models and BRT. The two well-known behavioral intention models, namely the Theory of Reasoned Action [64] and the Theory of Planned Behavior [65], have been developed to understand the determinants of behavior. These models mainly assume that behavior can be predicted by intentions, with global motives acting as predictors of intentions and beliefs serving as important antecedents of global motives. As a relatively new model of human behavior, BRT has been developed as an extension of these theories, which additionally considers the role of reasons [6]. Specifically, reasons are modeled as mediators between beliefs/values and global motives, where it is assumed that the way individuals process their belief and value information directly influences the reasons they provide for explaining their behavior. The details of the hypotheses and their relation to these theories are explained in the following sections. 

#### 3.2.1. Perceived Values and Global Motives

Schwartz [66] claims that values act as fundamental guides in individuals’ choices and assessments of behavioral options. Values are not objective assessments but rather the subjective perception of a consumer regarding a specific brand or product [57]. Values, unlike attitudes or beliefs, constitute an organized system and are usually regarded as determinants of attitudes and behaviors [67]. 

According to behavioral intention models and BRT, perceived values are expected to directly affect the global motives of consumers in their purchasing decisions [6]. This proposition is also consistent with many psychological models. 

According to the value-basis theory [68], attitudes are the result of a person’s more general set of values (see H1a). Results of an empirical study by Schultz and Zelezny [67] about predictors of environmental attitudes support the notion that values are linked with specific attitudes. In a more recent empirical study based on BRT [69], it has also been shown that environmental values that are in support of environmental protection have a positive impact on consumers’ green consumption attitudes. 

In the case of predicting subjective norm, the individual’s perceptions about specific normative referents may be related to the person’s perceived values [6]. Several recent empirical studies based on BRT ([70,71] among others) have also reported significant findings supporting the impact of values on both subjective norm (see H1b) and behavioral control (see H1c). 

Research on luxury consumption has also shown that luxury values are important predictors of attitude, behavioral control, and subjective norms [18,72]. For example, Loureiro and Araújo [18] examined the influence of individual and social luxury values on customer attitudes, perceived behavioral control, and subjective norms, as well as how these factors affect intentions to recommend and pay a premium for luxury clothing in the Brazilian market. The results indicate that individual values positively affect intentions, while social values positively influence subjective norms but negatively affect perceived behavioral control. 

In another conceptual study on luxury purchase behavior, Jain [72] proposed a conceptual framework based on the Schwartz value theory and TPB. The discussion of the relationships in this framework, which is based upon an extensive literature review, supports the link between values and global motives in luxury consumption. 

Based on these well-established theories and research on luxury buying behavior, it can be hypothesized that perceived values affect global motives.

**Hypothesis** **1.** *Perceived values positively affect global motives in jewelry purchasing behavior*.

**H1a.**  *Perceived values positively affect attitude in jewelry purchasing behavior*.

**H1b.**  *Perceived values positively affect subjective norm in jewelry purchasing behavior*.

**H1c.**  *Perceived values positively affect perceived behavioral control in jewelry purchasing behavior*.

#### 3.2.2. Moderating Effect of Reasons

This study suggests that, depending on how they are conceptualized, reasons have the potential to serve as moderators. Because of reasons’ multi-faceted influence on behavioral processes, the role of reasons can be examined from different theoretical perspectives. 

Previous studies on reason theory have investigated the motivational impact of reasons. One of these studies analyzed the effect of the reasons’ salience manipulation on subsequent behavior [73]. The research showed that context-specific factors (reasons) can overwhelm values, which clarifies the inconsistency issue between values and behavior. In other words, missing strong cognitive support (reasons) for values will reduce the impact of the values on behavior. Other studies have examined the moderating role of reasons on the attitude–behavior relationship [74,75]. These reason-based models generally hypothesize that an individual forms a favorable evaluation of a certain option when he/she has strong reasons aligned with his/her values and attitudes. 

In a more recent theory by Westaby [6], reasons are assumed to mediate the relationship between consumer beliefs and attitudes. In the same study, Westaby also examined the interaction effect of beliefs and reasons on global motives and intention. However, no significant results were obtained in the empirical study based on analysis of variance. Sahu et al. [7] point out in their review of BRT that only a few studies have examined the impact of moderating variables (especially for the relationship between values and attitudes), indicating a significant research gap in the existing literature on this topic (see also [6,31]). To partially address this gap, this research examines the moderating effect of reasons on the relationship between values and global motives. 

Another theoretical perspective useful in explaining the role of reasons is involvement theory. Involvement as a motivational concept is related to personal values and needs [76]. Consumers are more likely to be cognitively engaged and to actively evaluate reasons for high-involvement products [13]. Luxury goods, which are accepted as high-involvement products due to their high price, rarity, and complicated nature, require detailed research and knowledge [77]. The consumer is more likely to choose the decision alternative that is supported by strong reasons and aligns with his/her values, as they guide the reasoning and selection of decision alternatives. Reasons serve as context-specific factors that consumers use to justify and defend their judgements or intentions that affect their behavior. The interaction of strong reasons with personal values serves as a navigational tool in the consumer’s decision-making journey, shaping their perceived control over the behavioral outcomes associated with high-involvement product choices (see H2c). Consumers will be cognitively engaged and feel more confident and comfortable when buying luxury goods. Studies examining high-involvement products, including luxury goods, have used reasons such as price promotion [77], sign value, and rewarding [78] as moderating variables. 

It is essential to also address the conceptual difference between values and reasons in order to provide justification for the moderating role of reasons. Values have a broad scope and encompass various forms of thinking, whereas reasons specifically center on the cognitive processes individuals employ to rationalize their behavior [6]. For example, an individual who has never used jewelry to express herself or does not have hedonic feelings for jewelry may buy a jewelry as a wedding gift for a friend or relative because jewelry is a conventional gift for a wedding ceremony. In this example, the reason directly influences the person’s decision, while values may not always be a factor in the person’s decision (see also [14]). This suggests that consumers may engage in reasoning processes rather than solely relying on intuitive motives and values. Therefore, reasons may not necessarily come after perceived values temporally, as suggested in BRT (see also [17]). For example, consumers who share identical values may develop different attitudes due to their varying reasons for purchasing jewelry. In luxury consumption, the reasons are generally based on traditions, social culture, and self; therefore, they are considered as relatively consistent contextual variables (see [16]), making them promising candidates for a potential moderating factor. 

All the theories and models mentioned above have mainly examined the role of reasons with respect to values and attitudes (see H2a). The moderating effect of reasons regarding subjective norm and behavioral control has only been discussed by Westaby [6]. Therefore, this study aims to justify the moderating role of reasons between values and attitudes both theoretically and empirically, while also exploring the moderating effects between values and subjective norm as well as behavioral control. 

It is worth noting that, depending on how the phenomenon is conceptualized and studied, a variable may act as either a mediator or a moderator [79]. In some cases, it can be mathematically shown that the same variable may function as both a mediator and a moderator [80]. However, Karazsia and Berlin [81], along with other researchers, state that it is inappropriate to simultaneously analyze the moderator and mediator effects of the same concept assessed at the same time (see also the MacArthur approach). Hence, this study does not compare competing models where the concept of ‘reason’ is proposed as either a mediator or a moderator. Instead, it aims to provide insight into the moderator role of the reason.

Consequently, reason may be regarded as a moderator if the relationship between values and global motives varies significantly in terms of strength and/or direction across various levels of the reason. The following hypotheses are proposed accordingly.

**Hypothesis** **2.** *Reasons moderate the relationship between perceived value and global motives in the jewelry purchasing process*.

**H2a.**  *Reasons moderate the relationship between perceived value and attitude in the jewelry purchasing process*.

**H2b.**  *Reasons moderate the relationship between perceived value and subjective norm in the jewelry purchasing process*.

**H2c.**  *Reasons moderate the relationship between perceived value and perceived behavioral control in the jewelry purchasing process*.

#### 3.2.3. Global Motives and Intentions

One of the main objectives of behavioral intention models is the prediction of intentions. Both TPB and BRT have demonstrated the antecedent nature of global motives and their direct influence on intentions [6,62,82]. Research on luxury consumption has also shown that attitude, subjective norm, and perceived behavioral control directly impact intention [45,72].

In behavioral intention models, it is assumed that attitudes directly affect intentions in the consumer’s decision-making process (see H3a). Empirical studies in different fields, including luxury consumption, have supported this assumption [59]. As previously mentioned, purchasing luxury goods requires high involvement due to its characteristics, such as high price, rarity, and complicated nature. Research on consumer choice behavior has revealed a strong relationship between attitudes and purchasing decisions made regarding complex or expensive products with high levels of involvement and motivation [83]. Among the global motives, attitude has the highest level of explanation of intention [84].

Perhaps there is a consensus in the literature that one of the significant factors that can motivate consumers to purchase (masstige) luxury brands is the influence of referent groups [85]. Social relations and social pressures have a considerable impact on luxury consumption (see H3b). Apart from social recognition and acceptance within one’s salient social environment [86], imitating famous people also supports luxury consumption. The effort to resemble the upper class of society leads to luxury consumption. Moreover, when purchasing luxury products, where it is difficult to objectively evaluate quality and style, individuals tend to consider the opinions and thoughts of those they refer to. The social impact is relatively higher in brand preferences for complex, expensive luxury products that are rarely purchased and highly visible to others [87]. In a recent empirical study, Zhang et al. [45] explored the factors influencing Chinese consumers’ purchase intentions for luxury goods by extending the TPB. The findings indicate subjective norm had notable and direct effects on consumers’ purchase intentions. 

Luxury purchasing behavior, on the other hand, is a process where the consumer is willing to spend money and time to search and obtain it [88]. This relates to the concept of perceived behavioral control, which denotes an individual’s perception of how easy or challenging it is to carry out the desired behavior [62,82]. In several studies on luxury consumption, findings have revealed that the perceived behavioral control construct has significant and direct influences on consumers’ purchase intentions (see H3c) ([45,89,90] among others). 

As explained above, attitude, subjective norm, and perceived behavioral control directly influence the consumer’s purchase intention. Hence, the following hypotheses are proposed:

**Hypothesis** **3.** *Global motives positively affect intentions in the jewelry purchasing process*.

**H3a.**  *Attitude positively affects intentions in the jewelry purchasing process*.

**H3b.**  *Subjective norm positively affects intentions in the jewelry purchasing process*.

**H3c.**  *Perceived behavioral control positively affects intentions in the jewelry purchasing process*.

#### 3.2.4. Reasons and Intentions

Unlike the previous behavioral intention models, BRT suggests that the ability to predict intentions improves with the addition of reasons. Firstly, reasons activate the justification and defense mechanisms, which were not addressed by the behavioral intention models. Essentially, reasons can impact intention, as individuals tend to feel more confident when they have reasons to justify their behavior, even if their global motives may not quite align with their intentions. Secondly, further research has proven that there is a direct relationship between context-specific factors and intention. Context-specific factors that are ignored by traditional structures are captured through reasons. This finding aligns with the theory proposed by Davis et al. [91], which suggests that not all connections in behavioral intention models may become active in certain situations. Gigerenzer and Goldstein’s [92] research has also shown that reason-based decision-making serves as a shortcut for determining choices in real-life decision situations. Consequently, context-specific reasons that the consumer develops to justify his/her jewelry purchasing behavior and to justify his/her action may directly affect the intentions. The following hypothesis is proposed:

**Hypothesis** **4.** *In the jewelry purchasing process, reasons directly affect intentions without activating global motives*.

**Figure 1:**
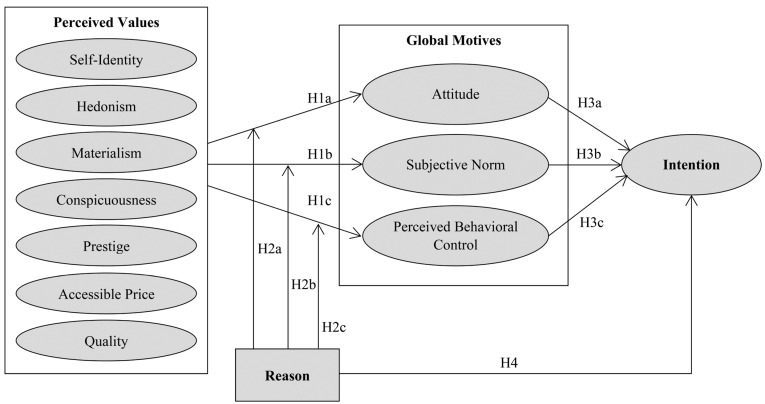
Proposed research model.

The proposed research model and the corresponding hypotheses are presented in Figure 1.

## 4. Methodology

In order to examine the proposed model and test the hypotheses, a quantitative approach based on PLS-SEM was employed using survey data on the consumption of masstige brands in the jewelry market in Türkiye. The steps of the empirical study are explained in the following sections.

### 4.1. Measures

An online questionnaire was designed for the survey which consisted of two sections. The first section of the questionnaire involved the measurement of the latent constructs, where respondents were instructed to consider their experience purchasing masstige jewelry. The second section of the questionnaire introduced the demographic questions, consisting of respondents’ gender, age, marital status, educational background, and occupation.

For the measurement of the constructs, common scales developed in the literature were adapted by adding original items or modifying existing ones. All constructs except reasons were measured using multiple-item scales. A 7-point Likert scale, ranging from 1 (strongly disagree) to 7 (strongly agree), was used to rate the items. 

The perceived value constructs and their indicators, tailored to the consumption of masstige jewelry, were partially adapted from the scales developed by Wiedmann et al. [21], Hennings et al. [52], Esmaeilpour [93], Park et al. [94], Shukla and Purani [86], Kim et al. [95], and Doss and Robinson [96]. Table 1 presents these constructs and their corresponding indicators. It is worth mentioning that two different applications are evident in the literature for the measurement of perceived values and their incorporation into structural equation models. Either multidimensional scales (i.e., second-order measurement models) [18,21,56] are used or perceived values are considered separately as single constructs (e.g., [52,86,97,98]). The latter is preferred in this study to provide a more comprehensive and clear explanation of the moderating effect (i.e., interaction effect of different combinations of values and reasons).

Reasons serve as important determinants of jewelry buying behavior, though they have not been used in the masstige marketing literature before. The scope of this study is limited to reasons that positively influence purchasing behavior to avoid additional complexity and focus on the moderator effect of reasons. As there has been insufficient research on the reasons that drive masstige consumption, a group of fifteen experts with over ten years of retail sales experience in the jewelry industry, specifically serving as jewelry store managers, were interviewed in an open-ended session lasting 90 min to generate possible reasons. They were requested to state the common reasons they frequently encountered that influence the purchasing behavior of masstige jewelry. The top seven reasons considered in this study are given in Table 2.

Note that instead of using a multi-item scale for Reason, each reason is handled as a single item to allow for a more comprehensive and detailed examination of the moderating role of reasons. To accomplish this, submodels representing various value–reason combinations are formed and analyzed, the details of which are presented in Section 5. Using single-item measures for reasons is not an uncommon practice (cf. [18,99]). As Westaby [6] argues, high reliability among reasons is not theoretically necessary in BRT, since individuals are likely to demonstrate significant diversity in their evaluations of the various reasons contributing to their behavior. Thus, each reason item will be considered separately in the model.

Indicators for measuring attitude towards jewelry purchase, subjective norm, and perceived behavioral control (see Table 3) were adapted from the scales proposed by Loureiro and Araújo [18], Cheng et al. [100], Das [101], and Esmaeilpour [93], which were specifically designed for the luxury market.

Finally, indicators related to intentions towards jewelry purchase (see Table 4) were adapted from Loureiro and Araújo [18], Zhang et al. [102], and Fishbein [103]. 

### 4.2. Sample and Data Collection

As mentioned above, an online survey was conducted using a self-administered questionnaire. Before the full-scale survey was launched, a pretest was performed with 27 respondents to check the wording, length, and consistency of the questionnaire, which may have affected the reliability and validity of the study. Accordingly, the wording of eight items were slightly modified. 

Following the revision of the questionnaire, the full-scale survey was conducted to examine the factors that influence the purchasing decisions of consumers who had bought jewelry (including diamond necklaces, rings, bracelets, and earrings) from the leading three masstige jewelry retail chains in Turkey within the last twelve months. To increase the representativeness of the survey, the data were collected from consumers who visited one of the 109 masstige jewelry stores located in the 16 cities with the highest jewelry consumption statistics [104]. While visiting the store, these consumers were given a link to the online questionnaire and asked if they would be willing to complete it. A total of 321 responses were received in one month, starting from June 2022. It was ensured that the number of respondents from each city was roughly proportional to the number of stores situated in that city. After excluding incomplete and inconsistent responses, 295 questionnaires were available for analysis. The sample size was considered adequate (to achieve a statistical power of 80% for detecting R^2^ values of at least 0.10 with a 5% significance level) and met the minimum sample size requirements for PLS-SEM as recommended by Cohen [105], Kock and Hadaya [106], and Hair et al. [107]. 

## 5. Analysis

### 5.1. Descriptive Statistics

The descriptive statistics presented in Table 5 describe the demographic characteristics, including gender, marital status, age, educational background, and employment status, of the 295 masstige jewelry consumers who participated in the survey. The survey data show that the majority of participants were female (65.1%), married (69.5%), and aged between 30 and 44 years (60.9%). A significant portion had university degrees (77.6%), and most were employed (77.6%). Some respondents did not disclose certain demographic information.

### 5.2. Structural Equation Modeling (SEM)

In this study, PLS-SEM is used, which enables the modeling of both observable indicators and latent variables within a relational and causal framework. PLS-SEM offers an effective estimation technique for simultaneously analyzing a set of distinct multiple regression equations [108]. The aim is to maximize the amount of variance explained in the dependent latent constructs. This approach is considered a good alternative to the covariance-based SEM (CB-SEM), as it overcomes various limiting assumptions. For instance, CB-SEM lacks the ability to yield consistent solutions when the distributions of the indicators deviate from normality. Furthermore, when the size of the sample is not sufficiently large, CB-SEM may either produce invalid outcomes or fail to converge [109]. 

To check whether the indicators were normally distributed, Shapiro–Wilk, Anderson–Darling, and Jarque–Bera tests were performed. The majority of the indicators did not meet the normality assumption, even after applying Box–Cox transformations. In addition, the sufficiency of the sample size was checked by examining the ratio of observations to estimated parameters. The results indicate a ratio slightly below 10:1. While there is no single universally accepted rule, the ratio appears to be below the threshold commonly considered as safe (see [110,111,112]). Since PLS-SEM provides accurate predictions even in situations where the sample size is small, the model contains many latent and indicator variables, and the indicators do not follow a normal distribution [107], the collected data were analyzed using PLS-SEM via SmartPLS 3.0 software.

### 5.3. Submodel Analyses and Moderator Effect 

In this empirical study, submodels were created to assess the impact of context-specific reasons on the value–global motives relationship in the masstige jewelry purchase process. These submodels were carefully selected based on the relevance of the reasons to the value–global motives relationship (see Table 6). For the evaluation of the measurement and structural models, one of these submodels, which focuses on the value ‘Hedonism’ (VHE) and the reason ‘to express emotions’ (REA7), will be thoroughly examined. Later, a summary of the results from the remaining submodels will also be presented to provide a more complete understanding of how well the hypothesized model structure fits the empirical data. In other words, the aim was not to compare these submodels; rather, it was to investigate whether the suggested hypotheses (H1–H4) found support or not across various combinations of values and reasons.

#### 5.3.1. Measurement Model

Measurement models, referred to as outer models within the context of PLS-SEM, represent the relationships between latent constructs and the corresponding indicator variables associated with them [113]. The study employed a reflective modeling approach to represent the latent variables, and the reflective measurement models were assessed using various metrics, including ‘Cronbach’s alpha’, ‘composite reliability’, ‘outer loadings’, ‘average variance extracted (AVE)’, and ‘heterotrait–monotrait ratio (HTMT)’. Recent research provides evidence indicating that the widely used Fornell–Larcker criterion shows a relatively limited ability to detect issues concerning discriminant validity [114]. Therefore, this criterion will not be reported in this study.

##### Outer Loadings

Despite following a regression-based approach, PLS-SEM is considered nonparametric and does not necessitate normal data distribution. This characteristic allows for the utilization of the non-parametric iterative sampling method known as bootstrapping. Bootstrapping is employed to determine the significance of the outer loadings. A critical *t*-value of 1.96, which indicates 5% significance level, was taken as the basis when evaluating the significance of the indicators. In the examined submodel, outer loadings of all indicators were found statistically significant (see Table 7).

A high outer loading on a latent variable (i.e., a high indicator reliability) indicates that the corresponding indicators share a considerable amount of common information captured by the latent construct. In most cases, a threshold of 0.70 is considered acceptable. Indicators with outer loadings ranging from 0.40 to 0.70 should be omitted from the scale only if removing them leads to an improvement in composite reliability. Only the outer loadings of GMPC2 and GMSN1 were less than 0.70, with the values 0.676 and 0.520, respectively (see Table 7). Since removing these indicators did not lead to improvements in the AVE and composite reliability values, they were consequently retained in the model. The remaining indicators demonstrate a strong association with the latent variable they are intended to measure. 

##### Internal Consistency Reliability

The Cronbach’s alpha values obtained to examine the internal consistency reliability of the latent constructs in the submodel all exceeded the acceptable threshold value of 0.70 [113], except for the subjective norm (GMSN) construct. Nevertheless, the subjective norm remained at an acceptable level with a value of 0.69, just slightly below the threshold. Table 8 provides the construct reliability values for the submodel, including the moderator effects (i.e., MOD: REA7 × VHE). 

Because of the weaknesses of Cronbach’s alpha reported in the literature (see [115]), the “composite reliability” metric is employed as an alternative for evaluating the internal consistency [113]. According to Hair et al. [113], composite reliability values ranging from 0.70 to 0.90 are considered satisfactory. In the submodel, composite reliability values ranged from 0.80 to 0.93 and were regarded as acceptable (see Table 8). 

Note that the construct reliability and convergent validity values of the single-item REA7 are all 1.00. This cannot be regarded as evidence that the measurement of REA7 is completely reliable. 

##### Convergent Validity

To ensure convergent validity, the external loadings of the indicators (as mentioned earlier) and the average variance extracted (AVE) were assessed. An AVE value of 0.50 or higher is required for convergent validity [116]. In the submodel, the AVE values were found to be higher than 0.50, indicating that the latent variable explains more than half of the variance observed in its corresponding indicators (see Table 8).

##### Discriminant Validity

To assess the discriminant validity of the latent constructs, HTMT [117] was used. Henseler et al. [117] propose a threshold of 0.90 for structural models that involve highly similar latent variables. A value close to or greater than 1 suggests potential issues with discriminant validity [109]. The HTMT values of the constructs in the considered submodel are given in Table 9. All HTMT values, except for the one between the constructs GMAT and INT, are close to or less than 0.90. As no HTMT values are near to or greater than 1, it can be concluded that discriminant validity has been achieved.

#### 5.3.2. Structural Model 

Once the reliability and validity of the measurement models have been established, the subsequent step involves analyzing the structural model to assess the degree of empirical support for the hypotheses and the underlying theory. This evaluation entails analyzing the explanatory capabilities of the model and examining the relationships among latent variables [113]. The commonly used evaluation criteria encompass path coefficients, significance values obtained through bootstrapping, and coefficients of determination [109].

##### Significance and Relevance of the Structural Model Relationships 

**Figure 2:**
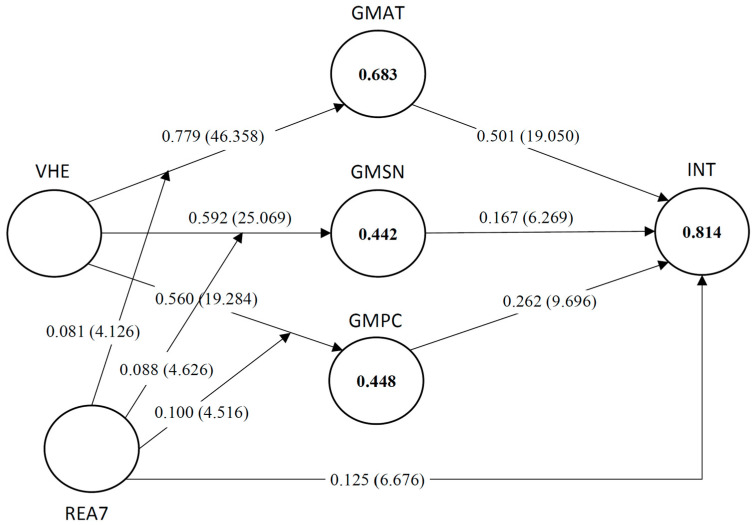
The standardized coefficients and *t*-statistics of the submodel, with moderator effect of the reason REA7 on the relationship between hedonism and global motives.

First, bootstrapping was employed to determine the significance of the path coefficients. In this empirical research study, 10,000 resamples were created for the calculation of *t*-statistics for all relationships defined in the submodel. The *t*-statistics computed for all path coefficients, including the moderator effect, exceeded 2.58, indicating that all relationships between latent variables differ from zero at a 1% significance level (see Figure 2). More specifically, the analysis of the submodel yielded a *t*-value of 4.126 for the path linking the interaction term (MOD1) and GMAT, a *t*-value of 4.626 for the path linking the interaction term (MOD2) and GMSN, and a *t*-value of 4.516 for the path linking the interaction term (MOD1) and GMPC. It can be concluded that REA7 has a significant moderating effect on the relationships between hedonism and the three global motive constructs.

The next concern is with the direction and size of the path coefficients as well as the effect sizes ($f^{2}$). Positive relationships with large effect sizes (refer to Cohen’s [118] definition) were found between hedonism and global motives (see Figure 2). To be more precise, an individual having hedonistic tendencies (VHE) feels the pleasure of buying and wearing jewelry, and this is reflected in his/her attitude towards purchasing (GMAT) (*β* = 0.779, *p* ≤ 0.01, $f^{2}$ = 1.574), which in turn influences his/her purchase intention (INT) (*β* = 0.501, *p* ≤ 0.01, $f^{2}$ = 0.545). Moreover, emotional reactions arising from buying masstige jewelry products (VHE) have a positive influence on the social pressure to purchase (GMSN) and the sense of control over the purchase behavior (GMPC) (*β* = 0.592, *p* ≤ 0.01, $f^{2}$ = 0.517; *β* = 0.560, *p* ≤ 0.01, $f^{2}$ = 0.468, respectively). A lower but significant impact was found on purchase intention from subjective norm (*β* = 0.167, *p* ≤ 0.01, $f^{2}$ = 0.066) and perceived behavioral control (*β* = 0.262, *p* ≤ 0.01, $f^{2}$ = 0.167). It was also observed that the path coefficients from REA7 to purchase intention were significant and positive with a small effect size (*β* = 0.125, *p* ≤ 0.01, $f^{2}$ = 0.066).

Examining the path coefficients of the moderating effects demonstrates a positive influence of the interaction term on GMAT (*β* = 0.081, *p* ≤ 0.01), GMSN (*β* = 0.088, *p* ≤ 0.01), and GMPC (*β* = 0.100, *p* ≤ 0.01) (see Figure 2). These results suggest that for higher levels of REA7, the relationship between hedonism and global motives increases by the size of the interaction term. For example, when REA7 is increased by one standard deviation unit, the relationship between hedonism and GMAT becomes 0.779 + 0.081 = 0.860, while it becomes 0.779 − 0.081 = 0.698 when REA7 is decreased by one standard deviation point. The effect sizes of the interaction terms MOD1, MOD2, and MOD3 were found to be 0.028, 0.018, and 0.024, respectively. Based on Kenny’s [119] findings for interaction terms, the values suggest a moderate effect size. The results provide strong evidence that REA7 has a significant and positive impact on the relationship between hedonism and global motives. 

##### Coefficient of Determination (R^2^ Value)

The coefficient of determination, R^2^, serves as a metric to assess the explanatory power of the model. When comparing models with varying numbers of explanatory variables, using the adjusted R^2^ metric is recommended [113,120]. The R^2^ values of the submodel are given in Figure 2. As seen in the figure, the R^2^ values of global motives and intention vary from 0.442 to 0.814, which indicates moderate to high levels of explanation of the latent variables. 

As the primary interest lies in the moderator effect, an additional PLS-SEM analysis was performed, excluding the moderator variable, and subsequently compared to the original model (including the moderator variable). The results demonstrate notable differences in the goodness-of-fit measures between the two models. The adjusted R^2^ values of the attitude, subjective norm, and perceived behavioral control in the model with the moderator variable (0.682, 0.441, and 0.446, respectively) are slightly higher compared to the model without the moderator (0.667, 0.419, and 0.407, respectively). This implies that for higher levels of the reason ‘to express emotions’ (REA7), individuals may be more influenced by hedonistic tendencies when forming attitudes, social norms, and beliefs about their ability to control their behavior in a given situation. On the other hand, those with lower REA7 might be less affected by hedonism in these aspects. 

Further model fit measures (SRMR, NFI, and RMS_Theta_) are provided in Appendix B.

### 5.4. Findings

The findings suggest that the inclusion of REA7 as a moderator improved the model’s explanatory power, supporting the hypothesis that reasons indeed moderate the relationships between values and global motives. Consequently, as indicated in Table 10, all hypotheses within the examined submodel were supported. 

Examining the results of the remaining submodels revealed similar findings for most of the value–reason combinations. The path coefficient estimates, *t*, and R^2^ values for each submodel can be found in Appendix C. In order to draw general conclusions about the proposed hypotheses, the results from all submodels were taken into account. 

Hypothesis 1 proposed that perceived values in jewelry buying behavior directly and positively influence global motives without the need for a causal intermediary. In the empirical study, it was observed that the path coefficients from values to global motives were statistically significant in all submodels, ranging from 0.186 to 0.779 for attitude, from 0.409 to 0.626 for subjective norm, and from 0.201 to 0.638 for perceived behavioral control (see Appendix C). These findings provide support for the hypotheses 1a, 1b, and 1c (see Table 11), indicating that perceived values indeed have a significant and considerable impact on global motives and are consistent with prior research.

Hypothesis 2 proposed that the reason involved in the process of purchasing jewelry would act as a moderator, influencing the relationship between perceived value and global motives. It was assumed that the moderator effect would offer a more comprehensive explanation of global motives. The obtained adjusted R^2^ values pointed to enhanced explanations of global motives and intention through the moderator effect, with an improvement ranging from 1 to 33% (see Appendix C). However, the empirical tests only partially supported Hypotheses 2a, 2b, and 2c (see Table 11). While most of the submodels produced results in favor of the hypothesis, there were three submodels where this was not observed. The combinations of materialism (VMA) and accessible price (VPR) with the reason ‘to take advantage of a campaign or promotion’ (REA6) yielded negative moderating effects for each global motive construct. This will be discussed in more detail later in the conclusion section. VSI-REA7 is another combination where the hypothesis was not supported and all moderator effects turned out to be insignificant (see Table 11). Moreover, within five submodels (VHE-REA3, VMA-REA4, VCO-REA1, VCO-REA2, and VPS-REA1), an insignificant moderator effect was obtained for one of the global motive constructs (either attitude or subjective norm). 

Hypothesis 3 posited that global motives have a positive influence on intentions within the context of jewelry purchasing. The empirical study revealed that the R^2^ value of intention exceeded 0.80 in all submodels, indicating a high level of explanatory power of intentions by global motives. Additionally, the *t*-statistics of the path coefficients from global motives to intention exceeded 2.58, indicating that all path coefficients were statistically significant at the 1% significance level (see Appendix C). Based on these findings, the hypotheses 3a, 3b, and 3c suggesting a positive relationship between global motives and intention were supported, and this result is in line with past models. 

Hypothesis 4 suggests that in the masstige jewelry buying process, reasons can directly affect intentions without involving global motives. Upon examining the results of the empirical analysis of the submodels, it was observed that the path coefficient representing the direct effect of the reason on intention varies between 0.030 and 0.170. Furthermore, except for two submodels (VCO-REA2 and VQU-REA2), the *t*-values of the coefficients were higher than 1.96, indicating that the path coefficients significantly differed from zero at the 5% significance level (see Appendix C). These findings provide support for Hypothesis 4, indicating that reasons indeed have a direct influence on the intention to purchase masstige jewelry and are consistent with BRT (Westaby, 2005 [6]).

## 6. Discussion and Implications

Below, a discussion of the findings is presented, along with their theoretical and practical implications.

### 6.1. Implications for Research

This study proposed a new model derived from BRT to examine the purchasing behavior of masstige jewelry. The suggested model offers a more comprehensive understanding of the determinants of purchasing masstige products by considering values and reasons in addition to the global motives and intention. Different from the original behavioral reasoning perspective, this study examined, for the first time, the moderating role of reasons. Reasons were considered as relatively consistent contextual variables, making them promising candidates for a potential moderating factor. The study explored how reasons may strengthen or weaken the impact of perceived values on global motives. It has been shown that, depending on how they are conceptualized, reasons have the potential to not only serve as mediators (as suggested in the literature) but also as moderators. As argued by Karazsia and Berlin [81], examining both the moderator and mediator effects of the same concept simultaneously is inappropriate. Therefore, any comparison of models in which the concept of ‘reason’ is introduced as a mediator in one and as a moderator in the other is avoided in this study. 

The results of the empirical study demonstrate notable differences in the goodness-of-fit measures between the moderated and non-moderated models. Specifically, the moderator effect of the reasons contributed to enhanced explanations of global motives and intention, with an improvement ranging from 1 to 33% within the analyzed submodels. All submodels except for two yielded positive moderating effects with reasonable effect sizes for each global motive construct. Nevertheless, not all submodels yielded significant results in terms of the moderator effect. Therefore, the empirical tests only partially supported the hypotheses regarding the moderating effect. 

When these findings are compared with related studies in the literature, there are both consistent and conflicting pieces of evidence. In his experimental analysis, Westaby (2005) examined BRT within the framework of employee turnover and relocation choices. He conducted an analysis employing ANOVA, which did not reveal any interaction effect (i.e., reason × belief) on global motives and intention. In our study, however, the moderating effect of reasons on the relationship between values and global motives (H2) has been observed to be significant across the majority of the submodels. The difference in statistical methods and items used, as well as the problem domain, may have an impact on the results. In this particular study, the outcomes also revealed that attitude exerted the most pronounced impact on intentions [6]. This finding aligns with Ajzen’s [62] assertion that attitudes consistently serve as robust predictors of intentions across various behaviors. In our study, we also arrived at the same finding. 

In another related study based on TPB, Loureiro and Araújo [18] investigated the relationship between values and global motives (H1) within luxury marketing, employing Wiedmann’s perceived value constructs. Their findings indicate that values serve as significant predictors of global motives. They categorized value dimensions into individual and social values, differing from the present research study. Their analysis revealed that individual luxury values significantly predict attitude, behavioral control, and subjective norms, while social values appear to exert a positive and significant influence on subjective norms and a negative impact on behavioral control. Specifically, the prestige value, categorized under social values in their study, demonstrated a negative effect on perceived behavioral control. However, contrary to their findings, in our study, the prestige value did not exhibit a negative impact on perceived behavioral control. 

Also, the findings obtained in the empirical studies conducted by Tani et al. [70] and Tani et al. [71] are in line with our findings for hypothesis H1. 

The remaining relationships (i.e., hypotheses H3 and H4) that have already been supported in the literature (e.g., Westaby [6]; Loureiro and Araújo [18]; Tani et al. [70]; Tani et al. [71]) were also supported in our empirical study, which is an important indicator for the reliability and validity of the study. 

Overall, the results indicate that introducing reasons as moderators in the model significantly enhances the understanding of global motives and intention, with positive moderating effects observed in the majority of submodels. Despite some inconsistencies with prior studies, the empirical findings are generally in line with the theories (reason theory, behavioral intention models, BRT, involvement theory, and value–basis theory) the hypotheses are based on. By examining the moderating effect of reasons on the relationship between perceived values and global motives, this study further extends the existing frameworks theoretically and offers a key contribution to the theory.

### 6.2. Implications for Practice

The empirical study relies on survey data concerning the consumption of masstige brands in the jewelry market in Türkiye, which has experienced a rapid growth over the past two decades. The current findings provide valuable insights into masstige jewelry purchasing behavior for marketers.

Understanding consumers’ luxury value perception and reasons for luxury consumption provides a better understanding of why they decide to purchase or avoid masstige goods. According to the descriptive results (mean), the values of hedonism (5.540) and quality (5.468) were rated the highest by consumers, implying that marketers should address the challenge of managing the balance between the quality, pleasure, and accessibility of their masstige brands (cf. [5]). The following most highly rated perceived values were self-identity (4.678) and materialism (4.626). Masstige luxury values closely align with traditional luxury values. Studies on traditional luxury values highlight hedonism [18], quality [121], self-identity, materialism [18], and prestige [21,122] as primary influencers of global motives, mirroring the first five masstige luxury values identified in this study. These parallel findings suggest that masstige consumers share comparable values and perceptions with their traditional luxury counterparts. 

The lower ratings for prestige and conspicuous consumption values among masstige consumers indicate that masstige jewelry is not perceived as prestigious as traditional high-end jewelry.

Price holds significance for traditional luxury consumers as a crucial luxury value [21,122], enhancing the desirability of luxury goods [86]. In the traditional luxury market, there is a greater desirability for high-priced goods, whereas in the masstige market, goods with an accessible price are less coveted. 

In addition, the results reveal that ‘leaving a beautiful memory/souvenir’ (5.998), ‘adhering to traditions’ (5.732), and ‘buying a gift for special occasions’ (5.336) were among the highest-rated reasons. These findings suggest that marketers should align their marketing strategies with these socially oriented reasons. Given the importance Turkish individuals place on traditional matters, such as ‘adhere to traditions’ and ‘leave a beautiful memory/souvenir’, these factors serve as crucial reasons.

The findings also show that while the attitudes, perceived norms, and perceived behavioral control of the consumers were partially explained by their values, the level of explanation improved when considering the moderating effect of reasons. In other words, for higher levels of the reasons, individuals were more influenced by luxury values when forming their global motives in purchasing masstige jewelry. This suggests that marketers can influence consumers with different values to buy for reasons that are relevant to their values. For example, in the submodel where pleasure and excitement arising from purchasing and wearing jewelry products were examined, it was observed that this value was reflected in the consumers’ global motives. If the consumer needs to buy a gift for a special occasion, this reason strengthens the influence of hedonistic satisfaction on her attitude toward and comfort in buying masstige jewelry. Marketers can use this information to their advantage by creating ads that associate these items with the hedonistic pleasure of celebration and emotional connection. This can help to encourage consumers who place a high value on pleasure to purchase and wear masstige jewelry when they have a special occasion coming up. Similar suggestions can be made for the reasons ‘adhere to traditions’, ‘reward oneself’, and ‘express emotions’ in combination with hedonistic satisfaction. 

In another submodel, the reason ‘adhere to traditions’ served as a significant moderator between both the values of quality and conspicuousness and the global motive of subjective norm. This indicates that people who value tradition are more likely to be influenced by the social pressure to purchase masstige jewelry that will make the individual stand out from the crowd. Moreover, it was observed that the attitude and purchasing comfort of a consumer who cares about quality and conspicuousness are strengthened by the moderating variable ‘to leave a beautiful memory/souvenir’. The consumer wants to make every moment unforgettable and leave a lasting impression with quality masstige jewelry.

On the other hand, the combinations of materialism (VMA) and accessible price (VPR) with the reason ‘to take advantage of a campaign or promotion’ (REA6) yielded negative moderating effects for each global motive construct. 

While masstige jewelry also holds considerable monetary value, materialism ranked as the fourth highest perceived value of masstige jewelry consumers in Türkiye. Materialistic individuals view jewelry as a sign of financial success, and when individuals with lower income can also access the same product during promotions, it becomes less appealing to them because it diminishes their uniqueness and no longer sets them apart from the general population. A similar phenomenon can be observed for the value of accessible price. When a product that was once considered prestigious or exclusive becomes widely available through campaigns or promotions, it loses its allure as a status symbol or marker of distinction. As more individuals can afford the product, it diminishes its ability to signal a higher social or financial standing (see also [52]). All these findings provide valuable insights for marketers in developing their communication strategies. 

## 7. Conclusions, Limitations, and Future Research

In this study, the moderator effect of reasons on the relationship between values and global motives was empirically examined for the masstige jewelry market in Türkiye. The contributions of this study can be summarized as follows:

- A new model derived from BRT was introduced that offers a more comprehensive understanding of the purchasing behavior of masstige products by considering values and reasons in addition to the global motives and intention.
- By analyzing consumer behavior in the growing masstige luxury market in Türkiye, particularly in the jewelry segment, the study contributed to the literature, where research is very limited.
- This study, for the first time, examined the moderating role of reasons and thereby extended the existing frameworks theoretically and offered a key contribution to BRT.
- There is limited research on the values that underlie luxury consumption, and no studies have specifically addressed reasons. This empirical study identified values and reasons that play a crucial role in purchasing masstige jewelry.
- The study employed statistical modeling to empirically test the proposed model using survey data on the consumption of masstige brands in the jewelry market in Türkiye, which has experienced rapid growth. The findings provide valuable insights into masstige jewelry purchasing behavior for both managers and researchers.

There are also some limitations and potential future directions that should be acknowledged. In a few submodels, the hypotheses related to the moderator impact were only partially supported. While this suggests some level of generalizability within the context of the masstige jewelry market in Türkiye, it is important to consider its limitations. In other words, generalizing these results to other luxury markets or regions may not be appropriate, as consumer behavior and market dynamics can vary significantly. Further research and replication studies in different contexts would be necessary to determine the broader applicability of the results.

As another limitation of this study, only reasons ‘for’ buying masstige jewelry were considered in the empirical analysis, despite the fact that BRT distinguishes between reasons ‘for’ and ‘against’ engaging in a behavior. Therefore, the effect of reasons against buying masstige jewelry, such as ‘economic instability’, ‘quality concerns’, and ‘lack of uniqueness’, can be investigated in future research. 

Moreover, each reason was considered as a single-item measure in the PLS-SEM. Therefore, developing and validating a multi-item measurement scale for reasons will be another topic for further research. 

Research has shown that past behavior is often directly predictive of future behavior without the need for components of behavioral intention models. Reasons are strengthened if behavior is consistently repeated [6]. In addition, Hair et al. [113] predicted that the reasons for repetitive behaviors, which were mediator effects before, can have a moderator effect over time. In this study, changes that occur with repeated purchasing (i.e., post hoc effects) were also not considered. Another focus for further research will be to examine the potential influence of reasons between past behavior and future behavior.

Numerous studies have explored the moderating effect of demographic characteristics on purchasing decisions. Future research could enhance the current model by incorporating these moderating effects.

Finally, the negative moderating effect of a few reasons that were expected to positively influence the value–global motives relationship during the empirical testing of the submodels should be further investigated through new empirical studies. 

## Figures and Tables

**Table 1:** Perceived value dimensions and indicators.

Constructs	Codes	Indicators	References
Self-Identification	VSI1	Masstige jewelry reflects the characteristics by which a person defines oneself.	Adapted from Wiedmann et al. [21], Shukla and Purani [86]
	VSI2	Masstige jewelry helps one express oneself.	
	VSI3	Masstige jewelry helps a person to explain oneself to others.	
	VSI4	The masstige jewelry worn gives an idea about the person.	
Hedonism	VHE1	Wearing masstige jewelry gives happiness.	Adapted from Wiedmann et al. [21], Shukla and Purani [86]
	VHE2	Buying masstige jewelry is an exciting experience.	
	VHE3	Wearing masstige jewelry is fun.	
	VHE4	Wearing masstige jewelry evokes good feelings.	
	VHE5	Wearing masstige jewelry makes you feel special.	
Materialism	VMA1	Masstige jewelry is a valuable tangible asset.	Adapted from Wiedmann et al. [21], Kim et al. [95]
	VMA2	Owning masstige jewelry is financially important to people.	
	VMA3	Masstige jewelry is an indicator of a financially successful life.	
	VMA4	Financially successful people wear masstige jewelry.	
	VMA5	People owning masstige jewelry are appreciated in society because of their financial situation.	
Conspicuous Consumption	VCO1	Wearing masstige jewelry is a sign of high social status.	Adapted from Park et al. [94]
	VCO2	Wearing masstige jewelry makes a strong impression on other people.	
	VCO3	Wearing masstige jewelry helps to attract attention in the environment.	
	VCO4	Wearing masstige jewelry is a sign of belonging to an elite class.	
Prestige	VPS1	Wearing masstige jewelry strengthens one’s image.	Adapted from Hennings et al. [52], Esmaeilpour [93]
	VPS2	Wearing masstige jewelry earns respect.	
	VPS3	Masstige jewelry has a positive social image.	
	VPS4	Wearing masstige jewelry leads to admiration by others.	
Accessible Price	VPR1	Masstige jewelry is worth the price paid.	Adapted from Hennings et al. [52]
	VPR2	The price to pay for the masstige jewelry is reasonable.	
	VPR3	The value of the masstige jewelry is worth the price paid.	
	VPR4	The cost of the masstige jewelry is reasonable alongside the benefits to be gained.	
Quality	VQU1	Masstige jewelry is durable.	Adapted from Doss et al. [96]
	VQU2	Masstige jewelry has attentive workmanship.	
	VQU3	Masstige jewelry has original design.	
	VQU4	Masstige jewelry has stones of high quality (properties).	

**Table 2:** Reasons.

Codes	Indicators
REA1	To adhere to traditions (on special occasions such as proposals, engagements, and weddings) by buying masstige jewelry.
REA2	To leave a beautiful memory/souvenir by buying masstige jewelry.
REA3	To buy masstige jewelry as a gift for special occasions such as Valentine’s Day, Mother’s Day, birthdays, promotion, and retirement.
REA4	To make an investment by buying masstige jewelry.
REA5	To reward oneself by buying masstige jewelry.
REA6	To benefit from of a campaign or promotion by buying masstige jewelry.
REA7	To express emotions (with symbolic signs) through buying masstige jewelry.

**Table 3:** Indicators suggested for global motives.

Constructs	Codes	Indicators	References
Attitude	GMAT1	I find it tempting to buy masstige jewelry.	Adapted from Cheng et al. [100], Das [101], Esmaeilpour [93]
	GMAT2	I like to buy masstige jewelry.	
	GMAT3	I think positively about buying masstige jewelry.	
	GMAT4	I find masstige jewelry attractive.	
Subjective Norm	GMSN1	When buying masstige jewelry, the opinion of my social environment is important for me.	Adapted from Cheng et al. [100], Loureiro and Araújo [18]
	GMSN2	My friends and family also approve of my masstige jewelry purchase.	
	GMSN3	Many of my friends also buy masstige jewelry.	
	GMSN4	The fact that people I like/admire wear jewelry affects my masstige jewelry purchase.	
Perceived Behavioral Control	GMPC1	I feel comfortable buying masstige jewelry.	Adapted from Loureiro and Araújo [18]
	GMPC2	I have sufficient knowledge about masstige jewels.	
	GMPC3	I do not feel uneasy when buying masstige jewelry.	
	GMPC4	I feel confident when buying masstige jewelry.	

**Table 4:** Indicators suggested for intentions.

Construct	Codes	Indicators	References
Intentions	INT1	I will recommend that others buy masstige jewelry as well.	Adapted from Loureiro and Araújo [18], Zhang et al. [102], Fishbein [103]
	INT2	I am thinking of purchasing masstige jewelry in the future.	
	INT3	I will advise my clients to buy masstige jewelry.	
	INT4	I want to buy masstige jewelry.	

**Table 5:** Demographic characteristics of participants.

Description		Ratio (%)	Sample Size
Gender	Female	65.1	192
	Male	32.2	95
	Not Mentioned	2.7	8
Marital Status	Married	69.5	205
	Not Married	28.1	83
	Not Mentioned	2.4	7
Age	Under 24	4.4	13
	25–29	15.6	46
	30–34	20.7	61
	35–39	21.6	64
	40–44	18.6	55
	45–49	12.9	38
	50–59	4.4	13
	60–69	1.0	3
	Not Mentioned	0.8	2
Educational Background	Primary School	1.3	4
	High School	20.3	60
	Graduate	55.9	165
	Post Graduate	21.7	64
	Not Mentioned	0.6	2
Employment	Employed	77.6	229
	Unemployed	18.7	55
	Not Mentioned	3.7	11

**Table 6:** Submodels.

		Reason						
Value		REA1	REA2	REA3	REA4	REA5	REA6	REA7
VSI	Self-identity							+
VHE	Hedonism	+		+		+		+
VMA	Materialism				+		+	
VCO	Conspicuousness	+	+					
VPS	Prestige	+						
VPR	Accessible Price				+		+	
VQU	Quality	+	+					

**Table 7:** Outer loadings and *t*-values of the indicators of the submodel VHE–REA1.

Code	Indicators	Outer Loadings	*t*-Values
VHE	VHE1	0.826	51.149
	VHE2	0.811	62.660
	VHE3	0.889	107.907
	VHE4	0.872	68.803
	VHE5	0.750	34.698
GMAT	GMAT1	0.898	116.448
	GMAT2	0.901	115.653
	GMAT3	0.908	113.271
	GMAT4	0.818	50.692
GMSN	GMSN1	0.520	15.431
	GMSN2	0.796	60.381
	GMSN3	0.787	48.777
	GMSN4	0.741	39.750
GMPC	GMPC1	0.785	50.387
	GMPC2	0.676	30.532
	GMPC3	0.829	56.584
	GMPC4	0.871	64.781
INT	INT1	0.870	90.007
	INT2	0.861	67.282
	INT3	0.884	99.531
	INT4	0.896	105.005

**Table 8:** Construct reliability and convergent validity.

Construct	Cronbach’s Alpha	Composite Reliability	AVE
VHE	0.887	0.917	0.690
GMAT	0.904	0.933	0.778
GMSN	0.690	0.808	0.518
GMPC	0.801	0.871	0.630
INT	0.901	0.931	0.771
MOD1 *	0.890	0.898	0.642
MOD2 **	0.890	0.918	0.693
MOD3 ***	0.890	0.915	0.630

*: between VHE and GMAT; **: between VHE and GMSN; ***: between VHE and GMPC.

**Table 9:** HTMT values of the submodel.

	GMAT	GMPC	GMSN	VHE	INT	REA7
GMPC	0.805					
GMSN	0.860	0.838				
VHE	0.907	0.741	0.794			
INT	0.942	0.903	0.902	0.878		
REA7	0.440	0.464	0.448	0.444	0.534	
REA7 × VHE	0.350	0.275	0.278	0.466	0.386	0.386

**Table 10:** Hypothesis testing for the VHE-REA7 submodel.

Hypothesis		*β*	*p*-Value	Decision
H1	Perceived values positively affect global motives in jewelry purchasing behavior.			
H1a	Perceived values positively affect attitude in jewelry purchasing behavior.	0.779	<0.01	Supported
H1b	Perceived values positively affect subjective norm in jewelry purchasing behavior.	0.592	<0.01	Supported
H1c	Perceived values positively affect perceived behavioral control in jewelry purchasing behavior.	0.560	<0.01	Supported
H2	Reasons moderate the relationship between perceived value and global motives in the jewelry purchasing process.			
H2a	Reasons moderate the relationship between perceived value and attitude in the jewelry purchasing process.	0.081	<0.01	Supported
H2b	Reasons moderate the relationship between perceived value and subjective norm in the jewelry purchasing process.	0.088	<0.01	Supported
H2c	Reasons moderate the relationship between perceived value and perceived behavioral control in the jewelry purchasing process.	0.100	<0.01	Supported
H3	Global motives positively affect intentions in the jewelry purchasing process.			
H3a	Attitude positively affects intentions in the jewelry purchasing process.	0.501	<0.01	Supported
H3b	Subjective norm positively affects intentions in the jewelry purchasing process.	0.167	<0.01	Supported
H3c	Perceived behavioral control positively affects intentions in the jewelry purchasing process.	0.262	<0.01	Supported
H4	In the jewelry purchasing process, reasons directly affect intentions without activating global motives.	0.125	<0.01	Supported

**Table 11:** Hypotheses testing results of all submodels.

	VSI-REA7	VHE-REA1	VHE-REA3	VHE-REA5	VHE-REA7	VMA-REA4	VMA-REA6	VCO-REA1	VCO-REA2	VPS-REA1	VPR-REA4	VPR-REA6	VQU-REA1	VQU-REA2	Overall
H1a	S	S	S	S	S	S	S	S	S	S	S	S	S	S	S
H1b	S	S	S	S	S	S	S	S	S	S	S	S	S	S	S
H1c	S	S	S	S	S	S	S	S	S	S	S	S	S	S	S
H2a	NS	S	S	S	S	NS	S	NS	S	NS	S	S	S	S	PS
H2b	NS	S	NS	S	S	S	NS	S	NS	S	S	NS	S	S	PS
H2c	NS	S	S	S	S	S	S	S	S	S	S	S	S	S	PS
H3a	S	S	S	S	S	S	S	S	S	S	S	S	S	S	S
H3b	S	S	S	S	S	S	S	S	S	S	S	S	S	S	S
H3c	S	S	S	S	S	S	S	S	S	S	S	S	S	S	S
H4	S	S	S	S	S	S	S	S	NS	S	S	S	S	NS	PS

S: supported, PS: partially supported, NS: not supported.

## Data Availability

The data presented in this study are available on request from the corresponding authors.

## Appendix A

**Table A1:** Dimensions and definitions of perceived value in luxury consumption.

Constructs	Definition	References
Self-identity	Self-identity refers to an individual’s inner self in terms of how he or she perceives himself or herself. The individual expresses himself/herself to the outside world by integrating the symbolic meaning of the luxury items into his/her own identity.	Wiedmann et al. [57]
Hedonism	Beyond functional utility, hedonism refers to emotional reactions such as perceived pleasure and reward, aesthetic beauty, and excitement arising from the purchase and consumption of luxury products.	Wiedmann et al. [57], Shukla and Purani [86]
Materialism	Materialism can be described as the importance of the possession in one’s life. The individual has tendency towards acquisition and assigns a high priority to material possessions.	Wiedmann et al. [57]
Conspicuousness	Conspicuousness refers to the practice of using products as symbolic representations to influence how others perceive oneself, thereby fulfilling social desires. The individual uses publicly visible luxury products for signaling social status, for differentiation, and for assimilation tendencies.	Wiedmann et al. [57], Schaefers [123]
Prestige	Prestige is a value that includes the respect that the product brings to its owner due to positive and successful perception. The use or display of a luxury product brings esteem for its owner.	Wiedmann et al. [57]
Accessible Price	Perceived price entails the consumer’s process of interpreting and assigning significance to price. Accessible price is an affordable price with reasonable premiums.	Kapferer et al. [124], Kapferer and Laurent [39]
Quality	Luxury products are associated with superior quality and a sense of reassurance, which results in higher value perceptions. In the context of luxury consumption, quality can be defined as emotional responses to design, craftsmanship, and durability.	Stylidis et al. [125], Vigneron and Johnson [22]

## Appendix B

Further fit measures of the structural model are given in Table A2. Since an SRMR value less than 0.08 is considered a good fit [126], the model indicates an acceptable fit. Moreover, the NFI values, which are close to 1, also indicate an acceptable fit. However, the RMS_Theta_ indicates a poor fit, since values below 0.12 represent a well-fitting model. Please note that these measures should be handled with care, as their potential contribution to PLS-SEM analyses in general remains an open question (see [113].

**Table A2:** Model fit measures.

Measure	Saturated Model	Estimated Model
SRMR	0.072	0.090
NFI	0.820	0.808
RMS_Theta_	0.210	

## Appendix C

**Table A3:** VSI–REA7 submodel—standardized path coefficients, *t*-statistics, and R^2^.

		Standardized Path Coefficients (*t*-Statistics)			Adj R^2^	
		VSI → GLBL MOT	GLBL MOT → INT and REA7 → INT	MOD	With MOD	Without MOD
Global Motives	GMAT	0.558 (19.152)	0.503 (19.941)	−0.087 (1.126)	0.435	0.402
	GMSN	0.613 (25.372)	0.163 (6.170)	0.049 (1.492)	0.449	0.437
	GMPC	0.537 (20.518)	0.263 (9.609)	0.064 (0.976)	0.413	0.381
Intention	INT		0.125 (6.404)		0.814	0.802

**Table A4:** VHE–REA1 submodel—standardized path coefficients, *t*-statistics, and R^2^.

		Standardized Path Coefficients (*t*-Statistics)			Adj R^2^	
		VHE → GLBL MOT	GLBL MOT → INT and REA1 → INT	MOD	With MOD	Without MOD
Global Motives	GMAT	0.741 (24.161)	0.501 (16.780)	0.067 (4.000)	0.680	0.668
	GMSN	0.535 (16.062)	0.170 (6.419)	0.079 (4.697)	0.443	0.419
	GMPC	0.495 (14.795)	0.278 (9.747)	0.115 (5.772)	0.449	0.408
Intention	INT		0.062 (2.645)		0.804	0.802

**Table A5:** VHE–REA3 submodel—standardized path coefficients, *t*-statistics, and R^2^.

		Standardized Path Coefficients (*t*-Statistics)			Adj R^2^	
		VHE → GLBL MOT	GLBL MOT → INT and REA7 → INT	MOD	With MOD	Without MOD
Global Motives	GMAT	0.707 (28.990)	0.463 (16.040)	0.057 (3.419)	0.689	0.668
	GMSN	0.469 (14.798)	0.139 (5.587)	0.061 (1.428)	0.470	0.419
	GMPC	0.494 (15.193)	0.093 (9.741)	0.093 (3.880)	0.449	0.408
Intention	INT		0.170 (7.939)		0.818	0.802

**Table A6:** VHE–REA5 submodel—standardized path coefficients, *t*-statistics, and R^2^.

		Standardized Path Coefficients (*t*-Statistics)			Adj R^2^	
		VHE → GLBL MOT	GLBL MOT → INT and REA7 → INT	MOD	With MOD	Without MOD
Global Motives	GMAT	0.688 (19.790)	0.498 (18.287)	0.074 (2.616)	0.690	0.668
	GMSN	0.553 (13.386)	0.176 (6.724)	0.091 (3.654)	0.438	0.419
	GMPC	0.520 (14.640)	0.283 (10.431)	0.091 (4.731)	0.433	0.408
Intention	INT		0.049 (2.192)		0.803	0.802

**Table A7:** VHE–REA7 submodel—standardized path coefficients, *t*-statistics, and R^2^.

		Standardized Path Coefficients (*t*-Statistics)			Adj R^2^	
		VHE → GLBL MOT	GLBL MOT → INT and REA7 → INT	MOD	With MOD	Without MOD
Global Motives	GMAT	0.779 (46.358)	0.501 (19.050)	0.081 (4.126)	0.683	0.667
	GMSN	0.592 (25.069)	0.167 (6.269)	0.088 (4.626)	0.442	0.419
	GMPC	0.560 (19.284)	0.262 (9.696)	0.100 (4.516)	0.448	0.407
Intention	INT		0.125 (6.676)		0.814	0.801

**Table A8:** VMA–REA4 submodel—standardized path coefficients, *t*-statistics, and R^2^.

		Standardized Path Coefficients (*t*-Statistics)			Adj R^2^	
		VMA → GLBL MOT	GLBL MOT → INT and REA4 → INT	MOD	With MOD	Without MOD
Global Motives	GMAT	0.455 (16.671)	0.505 (19.116)	−0.086 (1.235)	0.322	0.276
	GMSN	0.575 (22.967)	0.167 (6.182)	0.115 (2.156)	0.401	0.376
	GMPC	0.373 (12.256)	0.284 (10.058)	0.139 (2.384)	0.241	0.190
Intention	INT		0.094 (7.104)		0.809	0.802

**Table A9:** VMA–REA6 submodel—standardized path coefficients, *t*-statistics, and R^2^.

		Standardized Path Coefficients (*t*-Statistics)			Adj R^2^	
		VMA → GLBL MOT	GLBL MOT → INT and REA6 → INT	MOD	With MOD	Without MOD
Global Motives	GMAT	0.420 (15.328)	0.519 (20.364)	−0.162 (7.618)	0.388	0.276
	GMSN	0.504 (21.031)	0.151 (5.756)	−0.055 (1.110)	0.460	0.376
	GMPC	0.301 (10.870)	0.267 (9.066)	−0.145 (4.521)	0.344	0.190
Intention	INT		0.095 (5.613)		0.808	0.802

**Table A10:** VCO–REA1 submodel—standardized path coefficients, *t*-statistics, and R^2^.

		Standardized Path Coefficients (*t*-Statistics)			Adj R^2^	
		VCO → GLBL MOT	GLBL MOT → INT and REA1 → INT	MOD	With MOD	Without MOD
Global Motives	GMAT	0.274 (9.143)	0.503 (16.447)	0.047 (0.935)	0.435	0.227
	GMSN	0.444 (16.373)	0.166 (6.554)	0.079 (3.925)	0.446	0.344
	GMPC	0.240 (8.249)	0.281 (10.016)	0.154 (7.040)	0.371	0.178
Intention	INT		0.062 (2.653)		0.804	0.802

**Table A11:** VCO–REA2 submodel—standardized path coefficients, *t*-statistics, and R^2^.

		Standardized Path Coefficients (*t*-Statistics)			Adj R^2^	
		VCO → GLBL MOT	GLBL MOT → INT and REA2 → INT	MOD	With MOD	Without MOD
Global Motives	GMAT	0.186 (7.553)	0.508 (17.994)	0.083 (2.832)	0.556	0.227
	GMSN	0.420 (15.585)	0.176 (6.587)	0.052 (1.482)	0.437	0.344
	GMPC	0.201 (6.753)	0.288 (10.131)	0.079 (3.656)	0.370	0.178
Intention	INT		0.030 (1.243)		0.802	0.802

**Table A12:** VPS–REA1 submodel—standardized path coefficients, *t*-statistics, and R^2^.

		Standardized Path Coefficients (*t*-Statistics)			Adj R^2^	
		VPS → GLBL MOT	GLBL MOT → INT and REA1 → INT	MOD	With MOD	Without MOD
Global Motives	GMAT	0.439 (14.164)	0.503 (16.970)	0.092 (0.924)	0.533	0.388
	GMSN	0.503 (17.294)	0.166 (6.419)	0.082 (4.071)	0.483	0.410
	GMPC	0.327 (11.135)	0.282 (9.465)	0.204 (4.038)	0.424	0.255
Intention	INT		0.062 (2.770)		0.804	0.802

**Table A13:** VPR–REA4 submodel—standardized path coefficients, *t*-statistics, and R^2^.

		Standardized Path Coefficients (*t*-Statistics)			Adj R^2^	
		VPR → GLBL MOT	GLBL MOT → INT and REA4 → INT	MOD	With MOD	Without MOD
Global Motives	GMAT	0.670 (27.998)	0.504 (19.731)	−0.071 (2.587)	0.499	0.490
	GMSN	0.626 (25.382)	0.169 (6.510)	0.095 (4.416)	0.423	0.411
	GMPC	0.638 (26.491)	0.280 (10.266)	0.056 (1.995)	0.429	0.425
Intention	INT		0.093 (6.421)		0.809	0.802

**Table A14:** VPR–REA6 submodel—standardized path coefficients, *t*-statistics, and R^2^.

		Standardized Path Coefficients (*t*-Statistics)			Adj R^2^	
		VPR → GLBL MOT	GLBL MOT → INT and REA6 → INT	MOD	With MOD	Without MOD
Global Motives	GMAT	0.619 (24.055)	0.519 (19.794)	−0.152 (8.600)	0.551	0.490
	GMSN	0.533 (22.819)	0.153 (5.661)	−0.044 (0.692)	0.475	0.411
	GMPC	0.543 (22.154)	0.263 (9.703)	−0.104 (5.007)	0.499	0.425
Intention	INT		0.096 (5.487)			0.802

**Table A15:** VQU–REA1 submodel—standardized path coefficients, *t*-statistics, and R^2^.

		Standardized Path Coefficients (*t*-Statistics)			Adj R^2^	
		VQU → GLBL MOT	GLBL MOT → INT and REA1 → INT	MOD	With MOD	Without MOD
Global Motives	GMAT	0.385 (11.056)	0.501 (17.298)	−0.078 (3.479)	0.490	0.350
	GMSN	0.434 (13.742)	0.171 (6.476)	0.063 (3.608)	0.424	0.347
	GMPC	0.424 (12.908)	0.277 (9.678)	0.070 (2.917)	0.439	0.350
Intention	INT		0.063 (2.678)		0.804	0.802

**Table A16:** VQU–REA2 submodel—standardized path coefficients, *t*-statistics, and R^2^.

		Standardized Path Coefficients (*t*-statistics)			Adj R^2^	
		VQU → GLBL MOT	GLBL MOT → INT and REA2 → INT	MOD	With MOD	Without MOD
Global Motives	GMAT	0.266 (7.647)	0.505 (17.030)	0.081 (3.620)	0.576	0.350
	GMSN	0.409 (11.741)	0.178 (5.852)	0.083 (4.765)	0.422	0.347
	GMPC	0.386 (10.934)	0.284 (9.764)	0.077 (5.548)	0.439	0.350
Intention	INT		0.033 (1.346)		0.802	0.802
